# Impacts of hepatitis B and hepatitis C co-infection with tuberculosis, a prospective cohort study

**DOI:** 10.1186/s12985-020-01385-z

**Published:** 2020-07-23

**Authors:** Berhanu Elfu Feleke, Teferi Elfu Feleke, Wondimu Gebrekiros Adane, Abel Girma

**Affiliations:** 1 Department of Epidemiology and Biostatistics, University of Bahir Dar, Bahir Dar, Ethiopia; 2 Department of Pediatric, Butajira Hospital, Butajira, Ethiopia; 3 Private Health Sector Project, Addis Ababa, Ethiopia; 4 Department of Internal Medicine, University of Bahir Dar, Bahir Dar, Ethiopia

**Keywords:** Tuberculosis, Hepatitis B, Hepatitis C, Drug-induced hepatitis, Ethiopia

## Abstract

**Background:**

This study was conducted to estimate the prevalence, determinants of hepatitis B, hepatitis C and the survival of tuberculosis patients until drug-induced hepatitis.

**Methods:**

Prospective cohort study design was implemented. The data were collected from September 2016 – May 2019. Systematic random sampling was used to select the study participants. Baseline data were collected before the patient starts DOTS, the sign of liver toxicity was assessed every week. Tuberculosis treatment outcomes and WHO clinical stage was recorded at the end of 6th months. Descriptive statistics were used to estimate the prevalence of hepatitis B, hepatitis C viral infections and their effect on tuberculosis treatment outcomes. Binary logistic regression was used to identify the determinants of hepatitis B and C infections. The Kaplan Meier survival curve was used to estimate the survival of tuberculosis patient and Cox regression was used to identify the predictors of drug-induced hepatitis.

**Results:**

A total of 3537 tuberculosis patients were followed. The prevalence of hepatitis B and C viral infection among tuberculosis patients were 15.1 and 17.3% respectively. Hepatitis B viral infection among tuberculosis patients was associated with alcohol, sex, HIV, chronic illness. Hepatitis C viral infection among tuberculosis patients was associated with alcohol, sex, HIV, chronic illness. The incidence density for liver toxicity among tuberculosis patients was 843/15707 person-months and liver toxicity was determined by HIV, Hepatitis B, Hepatitis C, the severity of tuberculosis and chronic illnesses.

**Conclusion:**

Decision-makers should consider incorporating screening for hepatitis B and C viral infection during tuberculosis treatment.

## Background

Hepatitis is an inflammation of the liver cells called hepatocyte and, most commonly caused by hepatitis viruses. Hepatitis A hepatitis B, hepatitis C, hepatitis D, and hepatitis E viruses are responsible for injuring the hepatocyte. The severe form of hepatitis is caused by hepatitis B and hepatitis C viruses [1]. More than 2 billion people were infected with hepatitis B and annually killing 800,000 people [2–4]. The global prevalence of hepatitis C virus infection ranges from 2.5, − 3% [5, 6]. Globally, each year, 10 million new cases of tuberculosis and 1.6 million death of tuberculosis were reported by world health organization [7].

The prevalence of hepatitis B viral infection among tuberculosis patients ranges from 0.5 to 44% [8–11]. Hepatitis C virus burden among tuberculosis patients ranges from 3.4 - 44.6% [12–14].

In Ethiopia, 8% of the total population was infected with hepatitis B and 1.9% of the community was infected with hepatitis C virus [15, 16]. Ethiopia labeled as one of the high burden countries for tuberculosis [17]. The prevalence of smear positive tuberculosis was 108/100000 population and the prevalence of bacteriologically confirmed tuberculosis was 277/100000 population [18]. There is no evidence on tuberculosis hepatitis B and C co-infection rate in Ethiopia.

Hepatitis B or hepatitis C co-infection with tuberculosis increase the risk of treatment failure [19] activates latent tuberculosis [20–22], increase the risk of mortality [23], and drug-induced liver injury [24–28].

There is no updated evidence of tuberculosis, hepatitis B and hepatitis C co-infection in resource-limited setting like Ethiopia and the objectives of this research work were

1. To estimate the prevalence of hepatitis B viral infection among tuberculosis patients
2. To estimate the prevalence of hepatitis C viral infection among tuberculosis patients
3. To assess the determinants of hepatitis B and hepatitis C viral infections among tuberculosis patients
4. To describe the effects of hepatitis B and hepatitis C infection on tuberculosis treatment outcomes
5. To estimate the survival of tuberculosis patients until drug-induced hepatitis

## Methods

A prospective cohort study design was implemented among tuberculosis patients on directly observed treatment strategy (DOTS) following their treatment in West Gojam health facilities, Amhara regional state, Ethiopia. West Gojam is one of the 11 zones of Amhara regional state, Ethiopia. The data were collected from September 2016 – May 2019. Tuberculosis patients on DOTS were followed for 6 months. Transferred out tuberculosis patients and patient with incomplete medical records were excluded from the study.

Data were collected using an interview technique, document review, collecting the stool and blood samples. Clinical nurses were recruited to conduct the interview and extract necessary data from the medical records. Baseline data were collected before the patient start DOTS and the sign of liver toxicity were assessed every week. Tuberculosis treatment outcomes and WHO clinical stages were recorded at the end of 6th months. Laboratory technologists were recruited to analyze the stool and blood samples. For each tuberculosis patient, 1 g stool sample was collected and analyzed using concentration techniques to identify the presence of intestinal parasites. For each tuberculosis patient, 5 ml (ML) venous blood was collected using aseptic technique and Enzyme-linked immune Sorbent assay (ELIZA) test was performed to screen the presence of hepatitis B and hepatitis C. The standard operating procedures (SOP) were followed. CAGE tool was used to detect problematic alcohol use; viral load suppression was detected when HIV positive patient record less than 1000 copies of virus per ML of blood. Good treatments outcome was declared if the patient completed the treatments or become smear negative at the end of DOTS. To maintain the quality of the data: pre-test was performed on 50 tuberculosis patients, training was given for all data collectors and supervisors and the data collection procedures were closely supervised.

Epi-info software was used to calculate the sample size, the following assumptions were considered: 95% confidence interval, power of 90%, HIV positive to negative patient’s proportion of 1:3 and 10% loss to follow up rate. The estimated sample size was 912 HIV positive and 2731 HIV negative tuberculosis patients.

These samples were representative of tuberculosis population taking DOTS. The systematic random sampling technique was used to recruit HIV positive tuberculosis patients and HIV negative tuberculosis patients.

Epi-info software was used to enter the data and SPSS software was used for the analysis. Descriptive statistics were used to estimate the prevalence of hepatitis B, hepatitis C viral infections and their effects on tuberculosis treatment outcomes. Binary logistic regression was used to identify the determinants of hepatitis B and hepatitis C viral infections. The Kaplan Meier survival curve was used to estimate the survival of tuberculosis patient until drug-induced hepatitis. Predictors of drug induced hepatitis were identified using Cox regression.

Ethical clearance was obtained from Bahir Dar University ethical review board. Permission letter was obtained from Amhara national regional, state health bureau ethics committee and the respective health facility heads. Written informed consent was obtained from each study participant. The confidentiality of the data was kept at all stages. Study participants with hepatitis B or hepatitis C viral infections were linked to the available services in the health facilities.

## Results

A total of 3537 tuberculosis patients were followed, giving for a response rate of 97%, 106 tuberculosis patients were excluded from the study due to incomplete medical records and death. The mean age of the study participants was 34.49 years (standard deviation [SD] ± 15.6 years), 51.6% of the study participants were married and 80% of the study participants were from the rural areas (Table 1).

**Table 1:** Profile of the study participants (*n* = 3537)

Variables		Frequency	Percentage
Sex	Male	1734	49
	Female	1803	51
Resident	Rural	2817	79.64
	Urban	720	20.36
Educational status	Illiterate	170	4.8
	Informal education	707	20
	Formal education	2660	75.2
Problematic alcohol use	Present	383	89.2
	Absent	3154	10.8
Type of TB	Smear positive	996	28.2
	Smear negative	1782	50.2
	Extra pulmonary	759	21.5
Other chronic illness	Present	281	7.9
	Absent	3256	92.1
Smoking	Yes	301	8.5
	No	3236	91.5
IP^a^	Infected	2327	65.8
	Not infected	1210	34.2
HIV	Positive	2641	74.7
	Negative	896	25.3

^a^*IP* Intestinal parasite

The socio-demographic profiles of the whole study participants. The descriptions of 3537 tuberculosis patients were presented. The table also describes the proportions of Intestinal parasitic status infection and the HIV status of the tuberculosis patients

The prevalence of hepatitis B viral infection among tuberculosis patients was 15.1% [95% CI: 13.92 - 16.28%]. The prevalence of hepatitis B viral infection among HIV positive tuberculosis patients was 35.27%. The burden of hepatitis C viral infection among tuberculosis patients was 17.3% [95% CI: 16.06 - 18.55%], but this prevalence inflated to 46.09% of HIV infected tuberculosis patients.

After adjusting for residence, alcohol, smoking, sex, HIV, chronic illness, intestinal parasitic infections, and age; hepatitis B viral infection was associated with problematic alcohol use, sex, HIV, and chronic illnesses (Table 2).

**Table 2:** Determinants of hepatitis B viral infections among tuberculosis patients (*n* = 3537)

Variables		Hepatitis B		COR^**a**^ [95% CI]	AOR^**b**^ [95% CI]	***P***-value
		Positive	Negative			
HIV	Positive	316	580	6.06 [4.98–7.36]	4.95 [3.97–6.17]	< 0.01
	Negative	218	2423			
Sex	Female	494	1309	15.98 [11.48–22.24]	13.43 [9.53–18.93]	< 0.01
	Male	40	1694			
Chronic illness	Present	100	181	3.59 [2.76–4.68]	1.64 [1.2–2.22]	< 0.01
	Absent	434	2822			
Problematic alcohol use	Present	171	212	6.2 [4.93–7.79]	4.15 [3.03–5.67]	0.02
	Absent	363	2791			

^a^*COR* crude odds ratio

^b^*AOR* adjusted odds ratio

The predictors of hepatitis B viral infection among 3537 tuberculosis patients were presented. The binary logistic regression outputs for each predictor were presented using the adjusted odds ratio and *P*-values. Hepatitis B viral infection was associated with HIV, female sex, chronic illnesses, and problematic alcohol use

After adjusting for residence, alcohol, smoking, sex, HIV, chronic illness, intestinal parasitic infections, and age; hepatitis C viral infection was associated with problematic alcohol use, sex, HIV, and chronic illness. (Table 3).

**Table 3:** Determinants of hepatitis C viral infection among tuberculosis patients (*n* = 3537)

Variables		Hepatitis C		COR [95% CI]	AOR [95% CI]	***P***-value
		Positive	negative			
Problematic alcohol use	Present	142	241	3.36 [2.68–4.23]	1.46 [1.06–2.01]	0.02
	Absent	470	2684			
Sex	Female	527	1276	8 [6.29–10.19]	6.89 [5.25–9.04]	< 0.01
	Male	85	1649			
Chronic illness	Present	413	483	7.53 [5.84–9.72]	4.91 [3.65–6.6]	< 0.01
	Absent	199	2442			
HIV	Positive	413	483	10.49 [8.63–12.76]	10.26 [8.22–12.81]	< 0.01
	Negative	199	2442			

The determinants of hepatitis C viral infection among 3537 tuberculosis patients were presented. The binary logistic regression outputs for each predictor were presented using the adjusted odds ratio and *P*-values. Hepatitis C viral infection among tuberculosis patients were determined by problematic alcohol use, female sex, HIV, and chronic illnesses

The treatment outcome was good in 89.5% of hepatitis B negative TB patients, and among Hepatitis B positive patients the treatment success rate was 68.9%. The severity of tuberculosis presentation among hepatitis B positive TB patients was 80%. Good treatment outcome was observed in 92.6% of hepatitis C negative tuberculosis patients, and 56.9% of hepatitis C negative patients. The prevalence of severe tuberculosis was 58% among hepatitis C positive TB patients, and 23.5% among hepatitis C negative TB patients (Tables 4 and 5).

**Table 4:** The effect of hepatitis B viral infection on tuberculosis (*n* = 3537)

		Hepatitis B positive		Hepatitis B negative		X^**2**^	***P***-value
		Frequency	Percentage	Frequency	Percentage		
Treatment outcome	Good	368	68.9	2688	89.5	162	< 0.01
	Bad	166	31.1	315	10.5		
Retreatment category	Yes	250	46.8	251	8.4	552	< 0.01
	No	284	43.2	2752	91.6		
Severity of tuberculosis	Severe	427	80	616	20.5	771	< 0.01
	Not severe	107	20	2387	79.5		

The impacts of hepatitis B viral infection on tuberculosis were presented. The effects of hepatitis B viral infection on tuberculosis treatment outcome (Good, Bad), re-treatment category (Yes, No), and severity of tuberculosis (Severe, not severe) were presented using the X^2^ with its associated *P*-values

**Table 5:** The effect of hepatitis C viral infection on tuberculosis (*n* = 3537)

		Hepatitis C positive		Hepatitis C negative		X^**2**^	***p***-value
		Frequency	Percentage	Frequency	Percentage		
Treatment outcome	Good	348	56.9	2708	92.6	550	< 0.01
	Bad	264	43.1	217	7.4		
Retreatment category	Yes	198	32.4	303	10.4	201	< 0.01
	No	414	67.6	2655	89.6		
Severity of tuberculosis	Severe	355	58	688	23.5	290	< 0.01
	Not severe	257	42	2237	76.5		

The impacts of hepatitis C viral infection on tuberculosis were presented. The effects of hepatitis C viral infection on tuberculosis treatment outcome (Good, Bad), re-treatment category (Yes, No), and severity of tuberculosis (Severe, not severe) were presented using the X^2^ with its associated *P*-values

Hepatitis B and C significantly affected the efficacy of highly active anti-retroviral therapy (HAART); viral load suppression was 26.4% for hepatitis B positive TB patients, and 73.6% among hepatitis B negative TB patients. Among hepatitis C positive TB patients with viral load suppression were only 37.8%, but viral load suppression was 62.2% among hepatitis C negative TB patients (Tables 6 and 7).

**Table 6:** The effect of hepatitis B viral infection on HIV (*n* = 896)

		Hepatitis B positive		Hepatitis B negative		X^**2**^	***P***-value
		Frequency	Percentage	Frequency	Percentage		
Viral load	Suppressed	182	26.4	507	73.6	102	< 0.01
	Not suppressed	134	64.7	73	35.3		
WHO clinical stage	I.	189	30.2	436	69.8	45	< 0.01
	II.	51	36.7	88	63.3		
	III.	73	61.9	45	38.1		
	IV.	3	21.4	11	78.6		

The effects of hepatitis B viral infection on 896 HIV patients were presented. HIV positive viral load (suppressed, not suppressed), and world health organization clinical stages (stage I, stage II, stage III and stage IV) were cross checked with their hepatitis B viral infection status. X^2^ test and their corresponding *P*-values were used to check the associations

**Table 7:** The effect of hepatitis C viral infection on HIV (*n* = 896)

		Hepatitis B positive		Hepatitis B negative		X^**2**^	***P***-value
		Frequency	Percentage	Frequency	Percentage		
Viral load	Suppressed	279	40.5	410	59.5	38	< 0.01
	Not suppressed	413	46.1	73	35.3		
WHO clinical stage	I.	236	37.8	389	62.2	77	< 0.01
	II.	73	52.5	66	47.5		
	III.	94	79.7	24	20.3		
	IV.	10	71.4	4	28.6		

The effects of hepatitis C viral infection on 896 HIV patients were presented. HIV positive viral load (suppressed, not suppressed), and world health organization clinical stages (stage I, stage II, stage III and stage IV) were cross checked with their hepatitis C viral infection status. X^2^ test and their corresponding *P*-values were used to check the associations

**Fig. 1:**
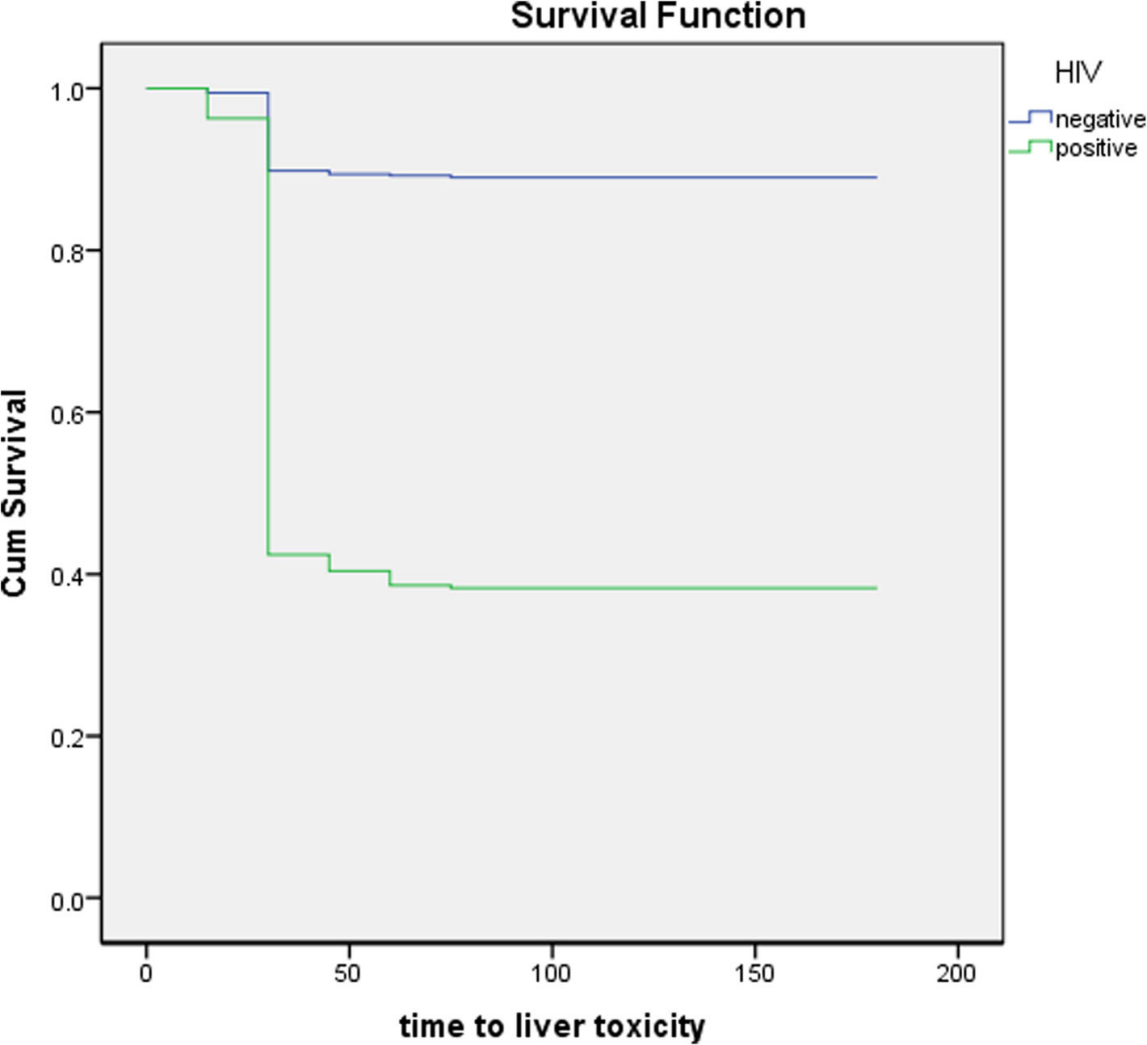
The risk of liver toxicity in HIV positive and HIV negative Tuberculosis patients during directly observed treatment strategy. The horizontal axis indicates the follow up time days and the vertical axis indicates the cumulative survival

The incidence density for the liver toxicity among tuberculosis patients was 843/15707 person-months. The median time of hepato-toxicity was 24 days (Fig. 1).

After adjusting for age, HIV, hepatitis B, hepatitis C, the severity of tuberculosis, chronic illness, smoking, and alcohol; hepatotoxicity was predicted by HIV, Hepatitis B, Hepatitis C, the severity of tuberculosis and other chronic illnesses (Table 8).

**Table 8:** Predictors of hepato-toxicity among tuberculosis patients (*n* = 3537)

Variables	B^a^	SE^b^	Sig.	Hazard ratio	95.0% CI for Exp(B)	
					Lower	Upper
HIV	.809	.092	.000	2.245	1.873	2.689
Hepatitis B	1.984	.086	.000	7.272	6.149	8.599
Hepatitis C	1.178	.083	.000	3.247	2.758	3.821
Severity of tuberculosis	.567	.083	.000	1.763	1.499	2.074
Chronic illness	.361	.088	.000	1.435	1.208	1.705

^a^*B* beta coefficient

^b^*SE* standard error

Cox regression outputs for the determinants of hepatotoxicity among 3537 tuberculosis patients were presented. The Hazard ratios with their corresponding *P*-values were presented for the predictors of hepatotoxicity among 3537 tuberculosis patients. Hepatotoxicity was associated with HIV, hepatitis B, hepatitis C, severity of tuberculosis and chronic illnesses

## Discussion

The odds of hepatitis B infection among HIV positive TB patients were 4.95 times higher; HIV infection increases the odds of hepatitis C viral infection by 10 folds. This finding agrees with a 2016 published systematic review [29]. This occurs because both hepatitis B and Hepatitis C virus share the same route of transmission with HIV/AIDS [30].

Hepatitis infection was higher in female, the odds of hepatitis B infection in female were 13.4 times higher and hepatitis C viral infection was 6.8 folds higher in the female. This finding agrees with other scholarly works [31, 32]. This is due to the anatomy and physiology of females reproductive organs which expose them to acquire these hepatitis viruses more easily [33, 34].

The presence of chronic illnesses increases the odds of hepatitis B by 1.6 folds and hepatitis C infection by 4.9 folds higher. This finding was in line with previous scholarly results [35–37]. This is because of the unhealthy lifestyle of the groups like substance abuse, unsafe sexual practices, and alcohol consumption habits which predisposes these patients to acquire the diseases [38].

Problematic alcohol use increases the odds of hepatitis B infection by 4 folds. The odds of hepatitis C viral infection were 1.5 folds higher among tuberculosis patients with problematic alcohol use. This finding was in line with previous work [39, 40]. This is because people with problematic alcohol use can commit unprotected sexual intercourse that exposes them to acquire the virus more easily [41].

Hepatitis B infection decreased the treatment success rate of tuberculosis by 20.6%; Hepatitis C viral infection decreased the treatment success rate of tuberculosis by 35.7%. This finding agrees with 2018 finding from China that indicates having hepatitis B or C decreased the treatment success rate [19]. This might be due to poor adherence of anti-TB drugs, poor bioavailability and metabolism of the drugs due to repeated vomiting as a result of hepatitis virus infections [42].

Hepatitis B infection increased the severity of tuberculosis by 59.5%, and hepatitis C increases the severity of tuberculosis by 34.5%. This finding agrees with previous findings [43]. This is because hepatitis viruses reactivate tuberculosis and lead to severe clinical presentations [44].

Viral load suppression was 47.2% lower among hepatitis B infected HIV patients and 19% lower among hepatitis C infected HIV patients. At the end of DOTS, 95% of the hepatitis C negative HAART patients had less than II WHO clinical stages, however, only 75% of hepatitis C infected HAART patients had less than II clinical stages. At the end of tuberculosis treatment, 76% of hepatitis B infected HAART patients had less than II clinical stages, but 90% of hepatitis B free HAART patient had less than II clinical stages. The finding was in line with the 2016 systematic review report [45]. This is because of the effects of hepatitis B and hepatitis C viral infection on the immune system of the host that leads them to the poor response to treatments [46, 47].

The incidence density for the liver toxicity among tuberculosis patients was 843/15707 person-months. This figure is higher as compared to the previous finding [48]. This might be due to the high alcohol consumption rate and adherence to traditional medicine in the study area.

HIV infection increases the hazard of liver toxicity by 2.25 folds. This result was in line with previously published findings [49]. This is due to the extra pill burden of HIV positive patients like Nevirapine, which causes additional hepatotoxicity [50, 51].

The risk of liver toxicity was 6 folds higher among hepatitis B infected DOTS patients, liver toxicity was 3 folds higher among hepatitis C infected tuberculosis patients. This finding agrees with finding from South Korea [24]. This is because the hepatitis virus accelerates the injury of liver cells as a result of anti-tuberculosis drugs [52].

The hazard of liver toxicity was 1.5 times higher in severe tuberculosis patients. This finding agrees with previous research work [53]. This might be due to the treatment regimen given to this group of tuberculosis patients with extra-anti-tuberculosis drugs.

Chronic illness increases the risk of liver toxicity by 1.2 folds during tuberculosis treatment. This finding was in line with other work [54]. This is because of the additional treatment given to the chronic diseases which increase the workload of the liver, causing additional injury to the liver cells [55, 56].

The limitation of this study was the failure to identify the specific serotypes for hepatitis B and hepatitis C viral infection. However, the main objective of this study was to identify the prevalence of all the serotypes and this limitation will not impose significant concern for this research.

## Conclusion

The prevalence of hepatitis B and hepatitis C viral infection was higher in tuberculosis patients. Hepatitis B and hepatitis C viral infection among tuberculosis patients were determined by HIV, alcohol, sex, and chronic illnesses. Hepatitis B and C co-infection lead to bad tuberculosis and HIV treatment outcomes.

## Recommendation

Decision-makers should consider screening for hepatitis B and hepatitis C viral infection to obtain good HIV and tuberculosis treatment outcomes.

## Data Availability

The datasets used and/or analyzed during the current study are available from the corresponding author on reasonable request.

## Abbreviations

AIDS: Acquired immune deficiency syndrome
AOR: Adjusted odds ratio
CI: Confidence interval
COR: Crude odds ratio
DOTS: Directly observed treatment strategy
ELIZA: Enzyme-linked immune Sorbent assay
HAART: Highly active anti-retroviral therapy
HIV: Human immune deficiency syndrome
ML: Milliliter
SD: Standard deviation
SOP: Standard operating procedures
TB: Tuberculosis
WHO: World health organization
